# Supplementary material for: The interactome of intact mitochondria by cross-linking mass spectrometry provides evidence for coexisting respiratory supercomplexes
Source: Mol Cell Proteomics. 2017 Dec 8;17(2):216–32. doi: 10.1074/mcp.RA117.000470 (PMC5795388; doi:10.1074/mcp.RA117.000470)
Supplement: Supplemental Data [file supp_RA117.000470_133922_0_supp_23978_fzffwf.zip › spectra_annotation/mito_spectra_annotation/11-1-6-1-7-1.pdf]

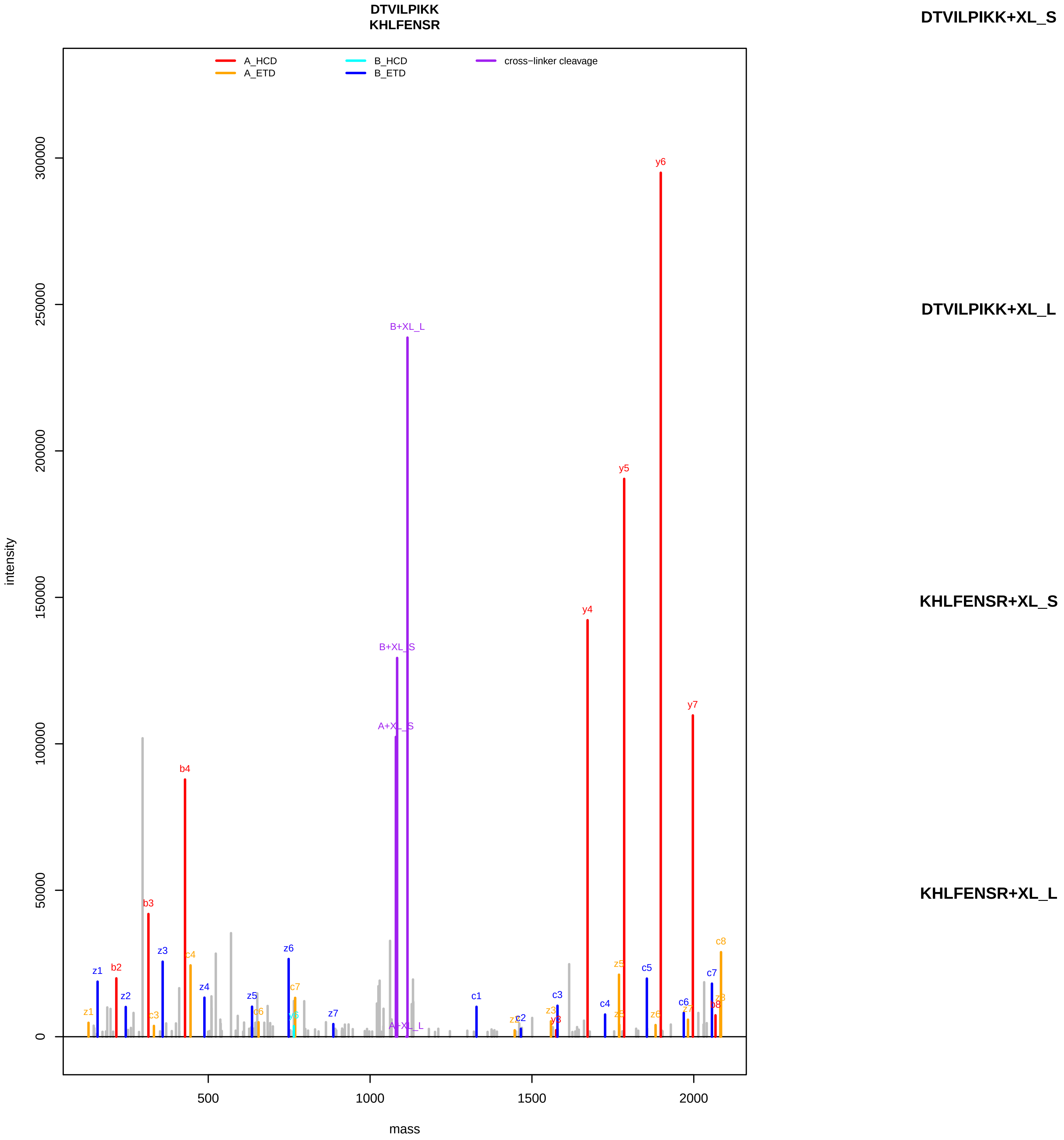
DTVILPIKK
KHLFENSR
A_HCD
A_ETD
B_HCD
B_ETD
cross−linker cleavage
intensity
mass
0
50000
100000
150000
200000
250000
300000
500
1000
1500
2000
y6
B+XL_L
y5
y4
B+XL_S
A+XL_S
y7
b4
b3
z1
b2
z2
z3
c4
z4
z5
z6
c7
z7
c1
c3
c4
c5
c7
c8
A+XL_L
DTVILPIKK+XL_S
DTVILPIKK+XL_L
KHLFENSR+XL_S
KHLFENSR+XL_L
